# Triboelectric Performance of Electrospun PVDF Fibers for Energy Harvesting: A Comparative Study of Boron Nitride (BN) and Reduced Graphene Oxide (rGO) Fillers

**DOI:** 10.3390/ma19030475

**Published:** 2026-01-24

**Authors:** Sunija Sukumaran, Piotr K. Szewczyk, Urszula Stachewicz

**Affiliations:** Faculty of Metals Engineering and Industrial Computer Science, AGH University of Krakow, 30-059 Krakow, Poland; sunija@agh.edu.pl (S.S.); pszew@agh.edu.pl (P.K.S.)

**Keywords:** PVDF, boron nitride, reduced graphene oxide, electrospinning, triboelectric nanogenerator, energy harvesting

## Abstract

The growing demand for smart electronic devices in daily life requires sustainable, renewable energy sources that reliably power portable and wearable systems. Triboelectric nanogenerators (TENGs) have emerged as a promising platform for smart textile-based energy harvesting due to their material versatility and mechanical compliance. In this work, electrospun poly (vinylidene fluoride) (PVDF) fiber mats incorporating boron nitride (BN) nanoparticles and reduced graphene oxide (rGO) were investigated to elucidate the roles of insulating and conductive nanofillers in governing the structural and electroactive properties of PVDF-based triboelectric materials. Electrospun PVDF mats containing 5 wt.% BN exhibited enhanced β-phase content (82%), attributed to the nucleating effect of BN and strong interfacial interactions between the nanofiller and the PVDF matrix. In contrast, 7 wt.% rGO demonstrated a high electroactive β-phase fraction (81%), arising from filler-induced dipole alignment and enhanced charge transport within the fibrous network. A comparative analysis of BN and rGO highlights filler-driven mechanisms influencing the electroactive phase formation and triboelectric charge generation in PVDF mats. The corresponding triboelectric power density reached 231 μWcm^−2^ for the 7 wt.% rGO/PVDF and 281 μWcm^−2^ for the 5 wt.% BN/PVDF-based TENGs, providing valuable insights for the rational design of high-performance, flexible triboelectric materials for wearable energy-harvesting applications.

## 1. Introduction

The rapid expansion of wearable electronics, smart textiles, and wireless sensor networks has intensified demand for lightweight, flexible, and sustainable energy sources that operate without external power supplies [1,2]. Triboelectric nanogenerators (TENGs) have emerged as a particularly attractive technology for harvesting low-frequency mechanical energy from ambient motions, owing to their simple device architecture, broad material compatibility, and high energy-conversion efficiency [3,4,5]. Unlike conventional electromagnetic or piezoelectric harvesters, TENGs can be readily integrated into deformable substrates and textile architectures, making them well-suited for next-generation self-powered systems [6,7].

Among triboelectric materials, poly (vinylidene fluoride) (PVDF) occupies a unique position due to its strong electronegativity, mechanical flexibility, and intrinsic electroactive behavior [8,9,10,11,12]. In addition to its triboelectric functionality, PVDF exhibits ferroelectric, piezoelectric, and pyroelectric responses when crystallized in its electroactive β-phase, enabling multifunctional energy harvesting within a single polymer system [10,13,14]. Electrospinning has proven to be a particularly effective route for PVDF processing, as the combined action of high electric fields and jet stretching promotes dipole alignment and β-phase formation without the need for post-poling treatments [15,16]. As a result, electrospun PVDF fiber mats have become a widely explored material system for wearable TENGs [16,17]. Regardless of the substantial progress, the triboelectric output of pristine PVDF remains limited by moderate surface charge density and charge dissipation during repeated contact-separation cycles [18,19]. To overcome these limitations, the incorporation of functional nanofillers has been extensively investigated as a strategy to tailor crystallinity, electroactive phase content, surface potential, and charge-trapping behavior [18,20,21]. Conductive carbon-based fillers, such as graphene oxide (GO) and reduced graphene oxide (rGO), have been shown to enhance β-phase formation via interfacial interactions and to improve charge transport and storage, resulting in significantly increased triboelectric output [20,22,23]. However, excessive electrical conductivity can also accelerate charge leakage, highlighting the need for careful balance between filler functionality and electrical insulation [18,24]. In contrast to conductive additives, electrically insulating fillers, such as boron nitride (BN), offer a fundamentally different mechanism for enhancing electrical performance [1,25]. BN combines high dielectric strength, chemical stability, and strong interfacial polarity, enabling effective nucleation of the PVDF β-phase while suppressing charge dissipation [26,27]. Recent studies have demonstrated that BN nanoparticles can serve as efficient charge-trapping sites, thereby enhancing surface charge density and improving triboelectric performance in PVDF-based nanogenerators [28,29,30]. Moreover, BN introduces additional multifunctionality by improving thermal stability and heat dissipation, which is particularly relevant for wearable devices operating under continuous mechanical loading [31,32,33].

While both conductive (rGO) and insulating (BN) fillers have independently shown promise in enhancing the electroactive and triboelectric properties of PVDF, a systematic comparison of their roles within identical electrospun architectures remains limited. In particular, the contrasting mechanisms by which conductive and insulating nanofillers influence β-phase formation and triboelectric charge generation remain incompletely elucidated. Addressing this gap is essential for rational material design, enabling the optimization of filler type and concentration for high-performance, flexible TENGs. Therefore, in this work, we investigate electrospun PVDF fiber mats incorporating rGO and BN nanofillers as representative conductive and insulating additives, respectively. By correlating morphological, structural, and electroactive characteristics with triboelectric output performance, we explain filler-dependent mechanisms governing charge generation and retention in PVDF-based TENGs. This comparative approach provides fundamental insights into the design of electrospun triboelectric materials and establishes clear guidelines for selecting nanofillers tailored to wearable and textile-integrated energy-harvesting applications.

## 2. Materials and Methods

### 2.1. Solution Preparation

PVDF solution preparation: A 24 wt.% Poly (vinylidene fluoride) (PVDF, Mw = 275,000 g/mol, Sigma-Aldrich, Gillingham, UK) solution was prepared by dissolving PVDF pellets in dimethylacetamide (DMAc, 99.8 % analytical grade, Avantor, Gliwice, Poland) and acetone ( 99.5 % analytical grade Avantor, Gliwice, Poland) in a 1:1 (wt./wt.) ratio. The solution was kept for continuous magnetic stirring at 650 rpm using a hot plate stirrer (IKA RCT basic, Staufen, Germany) at a constant temperature of 50 °C. The solution was stirred for 4 h until a homogeneous and transparent solution was obtained, following the previously reported protocol [8,22].

PA6 solution preparation: The polyamide 6 (PA6, Mw = 24,000 g mol^−1^, BASF, Ludwigshafen, Germany) was first dried to a constant weight at a temperature of 40 ˚C for 3 h. PA6 solution with a concentration of 12 wt.% was prepared by dissolving PA6 in formic and acetic acids in a 1:1 ratio (Avantor, Gliwice, Poland) with constant stirring at 400 rpm for 4 h on a hot plate (IKA RCT basic, Staufen, Germany) at an ambient temperature of 25 ˚C.

Composite solution preparation: Reduced graphene oxide (rGO) nanosheets, consisting of few-layer sheets (2–5 layers) with lateral dimensions of 1–10 µm and a thickness of 0.5–2 nm (Nanografi, Ankara, Turkey), and boron nitride (BN) nanoparticles (particle size < 150 nm, density 2.29 g cm^−3^; Sigma-Aldrich, Gillingham, UK) were used as fillers. The required amounts of rGO (7 wt.%) and BN (5 wt.%), corresponding to the PVDF concentration, were separately dispersed in the DMAc/acetone solvent mixture (1:1 wt./wt.) by ultrasonication (Emag, Emmi-E20, Mörfelden-Walldorf, Germany) for 4 h to ensure uniform dispersion and to avoid agglomeration. The resulting suspension was then individually added to the PVDF solution and magnetically stirred at 50 °C for 3 h. Finally, both composite solutions were subjected to an additional ultrasonication step of 2 h prior to electrospinning. The samples were labeled as 7wt.% rGO/PVDF and 5 wt.% BN/PVDF, respectively.

### 2.2. Electrospinning

Electrospinning of PVDF and composite fibers: Electrospinning of pristine PVDF and composite solutions was carried out using an electrospinning system (SKE, E-Fiber EF100, Bollate, MI, Italy) equipped with a climate control chamber. A stainless-steel needle with an outer diameter of 0.8 mm and an inner diameter of 0.5 mm was connected to a high-voltage power supply. PristinePVDF fibers were produced by applying a voltage of +20 kV, with a fixed needle to grounded collector distance of 18 cm and a solution flow rate of 1 mL h^−1^. For the 7 wt.% rGO/PVDF composite solution, an applied voltage of +20 kV was used, while the needle-to-collector distance was increased to 20 cm and the flow rate was set to 2 mL h^−1^. For the 5 wt.% BN/PVDF composite solution, electrospinning was performed at a higher applied voltage of +24 kV, with a needle-to-collector distance of 20 cm and a flow rate of 1 mL h^−1^. Electrospinning was carried out at a constant temperature of 25 °C and relative humidity (RH) of 30% for all samples.

Electrospinning of PA6: The electrospun PA6 fibers were produced by applying a voltage of 16 kV between the stainless needle and the grounded collector. The solution flow rate was set to 0.1 mL h^−1^ with a distance of 15 cm between the stainless needle and the collector. A constant temperature and RH of 25 ˚C and 40% were maintained throughout the electrospinning. The PA6 fibers were directly deposited on Al foil for 3 h of electrospinning time with a slow collector rotation (10 rpm).

### 2.3. Characterization of Electrospun Samples

Scanning Electron Microscopy (SEM): The surface morphology of the electrospun fibers was examined using a field-emission scanning electron microscope (Merlin Gemini II, Zeiss, Oberkochen, Germany) operated at an accelerating voltage of 3 kV and a working distance of 5.8 mm. Prior to imaging, all samples were sputter-coated with an ~8 nm thick gold (Au) layer using a rotary pump sputter coater (Q150RS, Quorum Technologies, Laughton, UK). Fiber diameter (D_f_) distributions were quantified from the SEM micrographs using ImageJ software (ver. 1.53v, National Institutes of Health, Bethesda, MD, USA). Elemental mapping was performed using energy-dispersive X-ray spectroscopy (EDS, Bruker, Oberkochen, Germany) to assess the distribution of nanoparticles within the composite fibers. Samples were gold-coated with a thin layer of Au (~8 nm) using a rotary pump sputter coater (Q150RS, Quorum Technologies, Laughton, UK). EDS mapping was conducted for 300 s at 10 kV and 1 nA with a working distance of 6 mm utilizing a backscattered electron detector.

Fourier transform infrared spectroscopy (FTIR): The relative content of the electroactive β-phase in PVDF and composite fiber mats was quantified using FTIR (Nicolet iS5 FT-IR spectrophotometer, Thermo Fisher Scientific, Waltham, MA, USA) spectroscopy. FTIR spectra were recorded in attenuated total reflectance (ATR) mode over the wavenumber range 600–1800 cm^−1^ with a spectral resolution of 1 cm^−1^, and the spectra were baseline-corrected before analysis. The β-phase fraction (F(β)) was calculated from the absorbance intensities of the characteristic β-phase band at 840 cm^−1^ and the α-phase band at 760 cm^−1^, following the established Lambert–Beer-based approach [14,34]. The β-phase fraction was determined using Equation (1):

(1) $$F(\beta )=\frac{A_{\beta }}{A_{\beta }+1.26A_{\alpha }}$$

where $A_{\beta }$ and $A_{\alpha }$ correspond to the absorbance values at 840 and 760 cm^−1^, respectively. Factor 1.26 accounts for the difference in absorption coefficients of the α and β crystalline phases. This method enables direct comparison of β-phase content between samples processed under different conditions or containing different fillers, while minimizing the influence of sample thickness and overall crystallinity [34].

Triboelectric energy harvesting: The triboelectric energy harvesting performance of PVDF, 7 wt.% rGO/PVDF, and 5 wt.% BN/PVDF fiber mats were evaluated using a bespoke setup equipped with a linear motor (LinMot P04, Lake Geneva, WI, USA). During the contact-separation mode (CS) operation of the TENG, the triboelectric-positive PA6 layer was periodically brought into contact with and separated from the triboelectric-negative PVDF-based mats. PVDF and composite mats (12 mm diameter) were mounted onto aluminum (Al) electrodes using conductive silver paste (Acheson Silver DAG 1415M, China). The PA6 counter electrode was prepared in an identical manner. The working parameters were applied at a frequency of 1.5 Hz under a contact force of 20 N, with a separation distance of 20 mm between the opposing electrodes. The short-circuit current was recorded using a high-precision electrometer (Keithley Instruments, LLC, Model 6517B, Cleveland, OH, USA). Additionally, the current and power outputs were measured across lower load resistors ranging from 0 to 100 MΩ for an effective TENG active area (A) of 1.13 cm^2^. Peak-to-peak current values were extracted and analyzed using OriginPro software (2022, OriginLab, Northampton, MA, USA). All measurements were performed on two independent samples for each material, and the reported current and power density values represent the averaged results. The power density $P_{A}$ was calculated according to the following equation [22,35].

(2) $$P_{A}=\frac{I^{2}R}{A}$$

where *I* is the short circuit current, *R* is the external load resistance, and *A* is the active contact area of the triboelectric device.

## 3. Results and Discussion

### 3.1. Morphology and Phase Analysis

In this work, we investigate the influence of electrically conductive and insulating nanofillers rGO and BN, respectively, on the crystalline phase composition and triboelectric performance of PVDF. A systematic concentration screening (1–10 wt.%) identified 7 wt.% rGO and 5 wt.% BN as the optimal loadings yielding the highest β-phase content for conductive and insulating fillers, respectively, from the previously reported study [8,22]. These compositions were therefore selected to enable a direct comparison of triboelectric behaviour.

**Figure 1:**
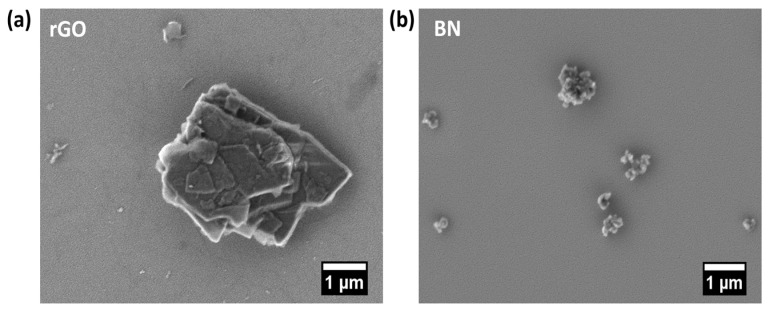
SEM micrographs showing the morphology of (**a**) rGO nanosheets and (**b**) BN nanoparticles.

The morphology of the fillers was examined by SEM to elucidate their structural characteristics prior to composite fabrication. As shown in Figure 1a, rGO exhibits a layered sheet-like structure, whereas BN nanoparticles (Figure 1b) display a spherical morphology with a particle size of < 150 nm, consistent with supplier specifications. These distinct filler geometries, combined with their contrasting electrical characteristics, electrically conductive rGO sheets, and electrically insulating BN particles, are expected to significantly influence polymer-filler interfacial interactions, jet stretching during electrospinning, and the resulting fiber morphology.

**Figure 2:**
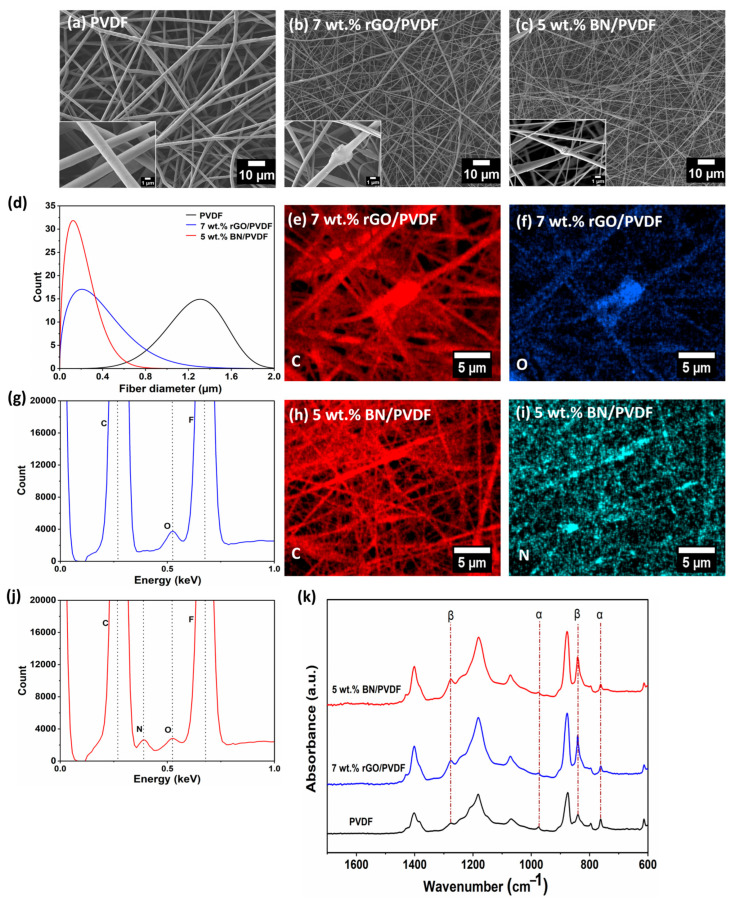
SEM micrographs illustrating fiber morphology, with a higher magnification inset showing the nanoparticle on an individual fiber. (**a**) PVDF, (**b**) 7wt.% rGO/PVDF, (**c**) 5wt.% BN/PVDF. (**d**) Fiber diameter distribution curve of PVDF, 7 wt.% rGO/PVDF, and 5 wt.% BN/PVDF. (**e**–**g**) EDS elemental mapping and corresponding spectrum of the 7 wt.% rGO/PVDF fibers showing C and O distributions. (**h**–**j**) EDS elemental mapping and corresponding spectrum of the 5 wt.% BN/PVDF fibers showing C and N distributions. (**k**) FTIR spectra of PVDF, 7 wt.% rGO/PVDF, and 5 wt.% BN/PVDF.

The surface morphology and microstructure of pristine PVDF, 7 wt.% rGO/PVDF, and 5 wt.% BN/PVDF composite fibers were characterized using SEM. Representative SEM micrographs of the randomly electrospun mats are displayed in Figure 2a–c, and the high magnification insets highlight the presence of nanoparticles on individual fibers. The PVDF fibers exhibit a continuous and bead-free morphology, achieved through optimization of the electrospinning parameters, as discussed in the Section 2. The corresponding fiber diameter distributions (D_f_) for all the samples are shown in Figure 2d. PVDF exhibited a relatively large average fiber diameter of 1.25 ± 0.28 μm, whereas a pronounced diameter reduction was observed upon incorporation of fillers. The 7 wt.% rGO/PVDF has an average fiber diameter of 320 ± 252 nm, followed by the 5 wt.% BN/PVDF fibers with an average D_f_ of 236 ± 142 nm. This substantial decrease in fiber diameter upon filler addition can be attributed to the following. Increasing the rGO or BN content increases the electrical conductivity and charge density of the polymer solution under an applied high electric field, leading to stronger stretching of the electrospinning jet [36,37,38,39]. As a result, rGO or BN/PVDF composite experiences more pronounced jet thinning, yielding a significant reduction in fiber diameter with increasing filler content [36,39]. Moreover, the smaller fiber diameter observed for 5 wt.% BN/PVDF can be ascribed to the lower particle size and more uniform dispersion of BN nanoparticles, which facilitates enhanced jet stretching during electrospinning compared to the larger, sheet-like rGO fillers. In addition, BN nanoparticle size is smaller than the fiber diameter, enabling their effective embedding within individual PVDF fibers. EDS mapping was employed to examine the distribution of nanofillers within the composite mats. As shown in Figure 2e–g, the elemental mapping and corresponding spectrum of oxygen (O) confirm the overall distribution of rGO within the 7 wt.% rGO/PVDF fibers. Similarly, the nitrogen (N) mapping in Figure 2h–j verifies the uniform distribution of BN nanoparticles in the 5 wt.% BN/PVDF composite. The corresponding spectra further validate successful filler incorporation into the PVDF matrix.

The triboelectric response of PVDF-based composite TENGs is closely governed by the proportion of electroactive crystalline phases [40]. Accordingly, the electroactive phases of the electrospun PVDF and composite fibers were examined using FTIR spectroscopy. As illustrated in Figure 2k, the absorption bands at 613, 763, and 946 cm^−1^ correspond to the non-electroactive α-phase of PVDF, in agreement with earlier studies [41,42]. The bands appearing at 840 and 1276 cm^−1^ are associated with the electroactive β + ϒ phases [40,43]. FTIR analysis reveals a marked enhancement of the electroactive β + ϒ phases’ intensity in 7 wt.% rGO/PVDF and 5 wt.% BN/PVDF compared with PVDF. In contrast, PVDF fibers display pronounced α-phase bands at 613, 763, and 946 cm^−1^, whereas these α-phases were substantially suppressed or nearly absent in the composite samples, indicating a phase transformation induced by 7 wt.% rGO and 5 wt.% BN by fillers incorporation. The relative β-phase fraction ($F_{\beta }$) was quantified using Equation (1) [34]. PVDF exhibits a β-phase content of 60%, while the β-phase content increased significantly upon filler addition, reaching 81% for 7 wt.% rGO/PVDF and 82% for 5 wt.% BN/PVDF. For the rGO incorporated fibers, the enhancement is ascribed to the dual role of rGO as a heterogeneous nucleation site and as a conductive filler that amplifies the local electric field during electrospinning, thereby promoting Coulombic stretching and alignment of PVDF chains into the all-trans β-phase conformation [37,44]. In the case of BN-filled PVDF, the higher β-phase fraction originates primarily from interfacial interactions between PVDF and BN. The polar B–N bonds in BN interact with the –CF_2_ dipoles of PVDF through dipole–dipole coupling, facilitating preferential β-phase crystallization despite the electrically insulating nature of BN [30,45]. Collectively, these results demonstrate that both conductive and insulating nanofillers can effectively promote electroactive phase formation in PVDF through distinct yet complementary mechanisms.

### 3.2. Triboelectric Performance

**Figure 3:**
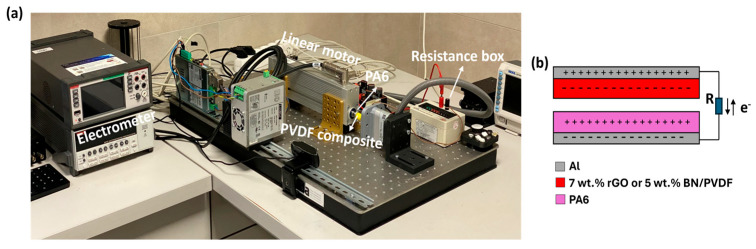
(**a**) Photographic image of the TENG measurement setup; (**b**) schematic illustration of the working mechanism of TENG operated in CS mode.

**Figure 4:**
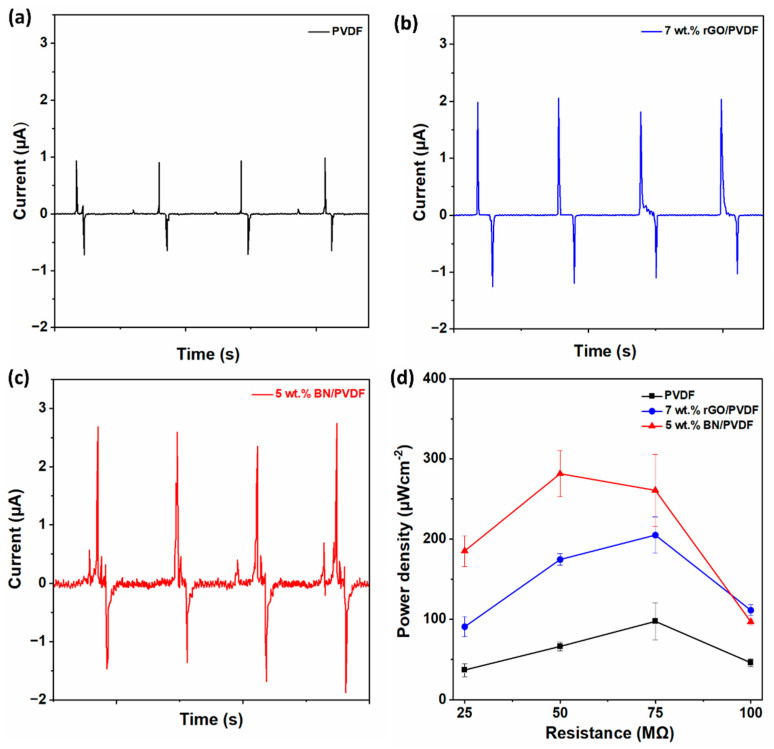
(**a–c**) Short-circuit peak-to-peak current output of PVDF, 7 wt.% rGO/PVDF, and 5 wt.% BN/PVDF TENG. (**d**) Power density across different load resistances.

Further, the triboelectric performance of electrospun PVDF, 7 wt.% rGO/PVDF, and 5 wt.% BN/PVDF mats were evaluated using a linear motor setup operated in CS mode (see Figure 3a). TENG consists of an electrospun nylon-6 (PA6) fiber serving as the tribo-positive counterpart, and Al foil served as the electrode for both triboelectric layers. Figure 4 summarizes the short-circuit current and power density outputs of the devices under identical operating conditions. The working mechanism of the TENGs was demonstrated in Figure 3b. During periodic contact separation between the tribo-negative PVDF mat (or 7 wt.% rGO and 5 wt.% BN/PVDF composite) and the triboelectric-positive electrospun PA6 layer, surface charge transfer occurs due to differences in their electron affinities. Upon contact, electrons are transferred from PA6 to the PVDF surface, generating opposite charges at the interface. Subsequent separation induces a potential difference between the Al electrodes, driving electron flow through the external circuit [22,46].

PVDF generated a short-circuit current of 1.3 μA (Figure 4a), which increased significantly upon filler incorporation. Figure 4b,c displayed the peak-to-peak short circuit current output from 7 wt.% rGO/PVDF and 5 wt.% BN/PVDF, respectively. The 7 wt.% rGO/PVDF composite delivered an enhanced current of 2.4 μA, while the 5 wt.% BN/PVDF device exhibited the highest short circuit current, reaching 3.0 μA at zero external load resistance. This increased current output correlates well with the improved electroactive β-phase content observed in the composite mats, which contributes to higher surface charge generation during triboelectrification.

The output power density (P_A_) further highlights the contrasting roles of conductive and insulating fillers (Figure 4d). The dependence of P_A_ on load resistance was determined using Equation (2). PVDF achieved a maximum power density of 97 μWcm^−2^ at an external load of 75 MΩ. In comparison, the 7 wt.% rGO/PVDF composite exhibited a markedly higher peak power density of 231 μWcm^−2^ at the same load resistance, corresponding to an enhancement of approximately 138% relative to pristine PVDF. The observed improvement occurs from enhanced charge generation due to the conductive pathways formed by rGO within the PVDF matrix, which accelerates efficient charge transport. Notably, the 5 wt.% BN/PVDF device delivered the highest power density of 281 μWcm^−2^, with the optimum output occurring at a lower load resistance of 50 MΩ, indicating improved charge retention and impedance matching. Compared to pristine PVDF, the power density increased by approximately 190% for the 5 wt.% BN/PVDF composite. This improvement is due to the efficient charge trapping and interfacial charge storage facilitated by BN [30,47], along with its high piezoelectric response and large surface area which enhance mechanical-to-electrical energy conversion [1,48]. Although the β-phase fraction is comparable for both composites (81–82%), the distinct triboelectric outputs are primarily governed by additive-driven differences in fiber morphology and filler dispersion. rGO and BN induce pronounced changes in fiber transverse area and surface roughness, which directly affect the effective contact area and surface charge density. Moreover, EDS mapping indicates a more homogeneous distribution of BN nanoparticles, which is expected to promote more uniform interfacial polarization/charge trapping and reduce local charge screening compared to larger rGO sheets.

Previous studies have demonstrated the effectiveness of graphene-based fillers in enhancing the triboelectric performance of PVDF-based nanogenerators. Dia et al. [23] reported a TENG fabricated from electrospun PVDF/Graphene oxide (GO) nanosheet, achieving an output current of 35 μA over a 25 cm^2^ device area and a maximum power density of 510 μWcm^−2^ at an optimized GO loading of 0.06 wt.%. Similarly, Shi et al. [20] developed a PVDF-graphene (G) nanosheet-based TENG and reported a substantially higher power density of approximately 13,020 μWcm^−2^ for a PVDF/1.5 wt.% graphene composite. Rana et al. [49] reported a PVDF/rGO film-based triboelectric nanogenerator, demonstrating that incorporation of 1.5 wt.% rGO resulted in a power density of ~95 μW cm^−2^ for a device area of 4 cm^2^. In a related study, the same group [50] developed electrospun PVDF composite fibers containing varying concentrations of nitrogen-doped reduced graphene oxide (N-rGO). Their results revealed that the PVDF/N-rGO composite with 1.5 wt.% filler content exhibited markedly enhanced triboelectric performance compared to conventional PVDF film-based TENGs, achieving a maximum power density of 282.8 μW cm^−2^ over an active area of 6 cm^2^. Beyond graphene-derived fillers, BN-based nanofillers have also been shown to significantly improve the triboelectric performance of PVDF-based systems. Zhang et al. [30] reported that incorporating 2 wt.% BN nanosheets into electrospun PVDF fibers resulted in ~600% enhancement in triboelectric signal amplitude compared to pristine PVDF, although the corresponding power output was not reported. Likewise, Yang et al. [1] demonstrated that the addition of a low loading (0.5 wt.%) of hexagonal BN nanosheets to PVDF nanofibrous mats led to a substantial increase in TENG performance, yielding a power density of 313 μWcm^−2^. Zhao et al. [29] demonstrated a high-performance and ultra-robust TENG based on hexagonal boron nitride nanosheets (hBNNS)/PVDF composite films, where inclusion of 2 wt.% hBNNS produced a power output of 4.84 mW in wind energy harvesting applications. Sahoo et al. [51] studied the effect of 2D nanofillers, including hBNNS and rGO, on the triboelectric performance of electrospun PVDF nanofibers. The authors reported that both rGO- and hBN-incorporated PVDF nanofibers exhibited enhanced triboelectric output compared to pristine PVDF, due to improved interfacial polarization, surface charge density, and filler-induced modulation of the electroactive β-phase. Notably, hBN addition was particularly effective in enhancing the triboelectric response due to its strong dielectric polarization and charge-trapping capability. However, explicit power output or power density values for the individual PVDF/rGO and PVDF/hBN composites were not separately reported in that work.

While several previously reported PVDF-based TENGs incorporating graphene or BN-derived fillers exhibit higher absolute power densities, these devices were typically evaluated using substantially larger active contact areas. In the present work, a compact device area of 1.13 cm^2^ was employed, which limits the total charge generation and absolute output. Nevertheless, the pronounced performance enhancement observed for both rGO- and BN-reinforced PVDF relative to pristine PVDF underscores the effectiveness of nanofiller-induced β-phase enhancement and triboelectric power generation even in small-area devices. In this context, the present work provides a direct, normalized comparison between conductive rGO and insulating BN fillers under identical device configurations, offering new insights into how charge generation governs triboelectric energy harvesting efficiency. These results highlight the suitability of the proposed material for compact, flexible, and scalable energy-harvesting applications.

## 4. Conclusions

In this work, the triboelectric energy harvesting performance of electrospun PVDF fiber was improved through the incorporation of conductive reduced graphene oxide (rGO) and insulating boron nitride (BN) nanofillers. Structural and electroactive phase analyses confirmed that both fillers effectively promoted the formation of electroactive β-phase in PVDF, increasing the β-phase fraction from ~60% in pristine PVDF to 81% and 82% for 7 wt.% rGO/PVDF and 5 wt.% BN/PVDF composites, respectively. Triboelectric characterization using nylon-6 as the tribo-positive counterpart revealed a substantial improvement in electrical output for both composite systems. The triboelectric power density increased from 97 μWcm^−2^ for neat PVDF to 231 μWcm^−2^ for 7 wt.% rGO/PVDF, corresponding to an enhancement of approximately 138%, while the 5 wt.% BN/PVDF composite achieved the highest output of 281 μWcm^−2^, representing a ~190% increase. Despite comparable β-phase contents, the superior performance of the BN-based composite highlights the decisive role of filler electrical nature in governing charge retention and minimizing dissipation during the contact separation mode of TENG operation. These findings highlight that controlling filler electrical characteristics is critical for optimizing power density in compact triboelectric nanogenerators, offering valuable design guidelines for next-generation flexible energy harvesting devices.

## Data Availability

The original contributions presented in this study are included in the article. Further inquiries can be directed to the corresponding author.
